# Real-Time Path Planning for Robot Using OP-PRM in Complex Dynamic Environment

**DOI:** 10.3389/fnbot.2022.910859

**Published:** 2022-06-09

**Authors:** Lingjian Ye, Jinbao Chen, Yimin Zhou

**Affiliations:** ^1^Shenzhen Institute of Advanced Technology, Chinese Academy of Science, Shenzhen, China; ^2^University of Chinese Academy of Science, Beijing, China

**Keywords:** path planning, narrow corridor, obstacle potential field, OP-PRM algorithm, incremental heuristic search algorithm

## Abstract

During task execution, the autonomous robots would likely pass through many narrow corridors along with mobile obstacles in dynamically complex environments. In this case, the off-line path planning algorithm is rather difficult to be directly implemented to acquire the available path in real-time. Hence, this article proposes a probabilistic roadmap algorithm based on the obstacle potential field sampling strategy to tackle the online path planning, called Obstacle Potential field-Probabilistic Roadmap Method (OP-PRM). The obstacle potential field is introduced to determine the obstacle area so as to construct the potential linked roadmap. Then the specific range around the obstacle boundary is justified as the target sampling area. Based on this obstacle localization, the effectiveness of the sampling points falling into the narrow corridors can be increased greatly for feasible roadmap construction. Furthermore, an incremental heuristic D* Lite algorithm is applied to search the shortest paths between the starting point and the target point on the roadmap. Simulation experiments demonstrate that the OP-PRM path planning algorithm can enable robots to search the optimal path fast from the starting point to the destination and effectively cross narrow corridors in complex dynamic environments.

## 1. Introduction

Trajectory planning refers to searching for available paths from the starting point to the target point while satisfying the constraints in a complex environment. Recently, trajectory planning for robots has become a research hotspot (Aggarwal and Kumar, 2020), especially for autonomous task execution in unknown environments.

The commonly used trajectory planning algorithms include sampling-based Probabilistic Roadmap (PRM) (Lin and Saripalli, 2017; Patle et al., 2019), Rapidly exploring Random Tree (RRT) (Zhang et al., 2020) and their variants PRM* (Palmieri et al., 2019), and RRT* (Wang et al., 2020), search-based A*, D* (Ab Wahab et al., 2020). The typical method to solve the trajectory planning is to grid the configuration space so as to construct the grid map with the occupied grids and the free grids, where the adjacent free grids are connected as the feasible target planning space. A search algorithm, such as A*, is normally applied on this type of map to search the available paths. However, the limitation of the grid map is that the rotation angle of the robot is discrete (Kala, 2019).

The roadmap is a kind of discrete graph contained in the discontinuous configuration space. After generating the roadmap, the graph search technology can be used for path planning, which is related to the shape of the obstacles and the dimension of the configuration space. The robot path planning problem can be expressed as finding a path τ[0, 1] → *C*_*free*_ from the current starting point *S* → τ(0) ∈ *C*_*free*_ to the target point *G* → τ(1) ∈ *C*_*free*_, where *C*_*free*_ represents the free area in the configuration space *C*, and the obstacle area is represented as *C*_*obst*_, *C* = (*C*_*free*_∪*C*_*obst*_). In the path planning, the roadmap *R*(*V, E*) is constructed, where *V* is the vertex set and *E* is the edge set that connects the vertices. The roadmap is usually constructed offline with a sampling strategy, i.e., random sampling, Halton points, Gaussian sampling, and obstacle-based sampling (Wang et al., 2019), while the search algorithm is used to find the path online when querying (Debnath et al., 2019).

The general sampling strategy is random uniform sampling to sample points in the *C*_*free*_ area. Since the volume of *C*_*free*_ in a dense obstacle environment takes a small proportion of *C*, the generation of effective sampling points in narrow corridors largely depends on setting a higher limit of sampling area to reduce the calculation cost. Such representative robot path planning algorithms include basic PRM and lazyPRM (Bohlin and Kavraki, 2000), the latter is less likely to generate samples in narrow corridors. In the obstacle-based sampling strategy (Amato et al., 1998), a sample *q*_*obst*_ is first generated in *C*_*obst*_, and sample *q*_*free*_ is generated in *C*_*free*_, while *q*_*free*_ is moved toward *q*_*obst*_ until it crosses the obstacle boundary so as to generate an effective sample *q*_*boundary*_ to be added to the vertex set. However, narrow corridors take a small region of the *C*_*free*_ among *C*_*obst*_, thus the sample migration method of *q*_*free*_ moving to *q*_*obst*_ does not always increase the effective sampling points in narrow corridors. Another improved mechanism is that *q*_*obst*_ moves in random directions until it reaches the obstacle boundary to generate an effective *q*_*boundary*_ as a new sample.

Extensive studies have been carried out *via* sampling-based methods to deal with path planning with narrow corridors in dense obstacle scenarios. Denny and Amatoo (2013) have proposed a Toggle PRM to introduce sampling in the obstacle configuration space. However, it is time consuming to maintain a large number of effective sampling points in both the obstacle and free spaces, not to mention large storage demand. Tahir et al. (2018) introduced a new concept of potentially guided bidirectional trees in the intelligent RRT* algorithm, called PIB-RRT*, thus greatly improving the convergence rate and memory utilization. Wang et al. (2020) proposed an optimal path planning algorithm based on the convolutional neural network, namely the neural RRT*, which can use the A* algorithm to generate the training data set consisting of the map information and the optimal path, gaining the balance between effectiveness and efficiency. Saha et al. (2005) refined and thinned the robot itself to indirectly increase the width of the corridor. However, this method does not pay attention to the sampling technique of the narrow corridor. Another widely adopted mechanism is to use a hybrid sampling technique, with a set of samplers, each of which can generate certain samples. Vonásek et al. (2020) proposed using multiple samplers simultaneously, and the contribution of each sampler is determined by the connectivity. With the same principle, Tsardoulias et al. (2016) divided the configuration space into various areas using different samplers based on the obstacle density and connectivity. Kannan et al. (2016) also used hybrid sampling technology to make the roadmap generation process conscious of almost all possible isomorphic path generation.

In addition, strategy can be applied to uniformly sampling in the obstacles neighborhood (Bera et al., 2014). With the Gaussian sampling strategy, a sampling point can be generated in *C*_*obst*_, while the acquired other sampling point within a specific distance, is followed by the Gaussian distribution with zero means (Boor et al., 1999). However, the possibility of generating samples near obstacles is quite low. As for the bridge test sampling strategy (Hsu et al., 2003), an invalid sampling point is first generated in *C*_*obst*_ to connect to another generated invalid sampling point, where the midpoint can be added in vertex set *V* if it falls into the *C*_*free*_ space. This method is similar to “bridging” among obstacles to increasing the number of effective sampling points but with fewer sampling points generation in a narrow corridor to save more calculation time. The dual roadmap can generate roadmaps *R*_*free*_ and *R*_*obst*_ in *C*_*free*_ and *C*_*obst*_ respectively at the same time (Kala, 2019), which does not limit the width of narrow corridors but can generate effective sampling points in any ordinary corridor. At present, there is seldom literature that investigates real-time path search based on a roadmap. Allen and Pavone (2019) proposed a new method for kinematic and dynamic motion planning in a random roadmap containing static and moving obstacles. However, this method is not suitable for real-time application and more time is required for path planning.

Search-based methods are also commonly applied for path planning on the constructed roadmap (Aine and Likhachev, 2016), as listed in Table 1. Given the known global map of the static environment, search methods seem less important. In dynamic environments, however, with unknown or mobile obstacles, reverse search combined with incremental search is more effective. For example, D* (Xue et al., 2014) and D* Lite (Koenig and Likhachev, 2005) are dynamic incremental algorithms extended from A* (Sudhakara and Ganapathy, 2016; Le et al., 2018). D* Lite algorithm can dynamically search the shortest path on the grid map, and use the point distance generated in the previous iteration to constantly update the optimal path from the current point to the target point (Koenig and Likhachev, 2005). They can reuse the previous searched information to speed up the current search, and its implementation is fast enough to play an important role in the real-time planner (Yang et al., 2016; Amarat and Zong, 2019). Jump Point Search (JPS) algorithm is also extended from A*, which identifies and selectively expands only certain nodes in a grid map called jump points (Harabor and Grastien, 2011). JPS expands the successor nodes based on the strategy of searching jump points, which makes it have better real-time performance than A*.

**Table 1:** Performance comparison of the search algorithms:Dijkstra (Wang et al., 2011), A* (Le et al., 2018), D* (Xue et al., 2014), LPA* (Koenig et al., 2004), and D* Lite (Koenig and Likhachev, 2005).

**Algorithm**	**Search direction**	**Heuristic**	**Incremental**	**Applicable scenarios**	**Practical applications**
Dijkstra	Forward	No	No	Global known information, static planning.	Selection of the shortest route in the network communication
A*	Forward	Yes	No	Global known information, static planning.	ApollGames, Robot path planning
D*	Backward	No	Yes	Partial known information, dynamically programmable.	Robot pathfinder, Mars rover path planning
LPA*	Forward	No	Yes	Partial known information, assumed dynamic unknown free space.	Robot path planning
D* Lite	Backward	Yes	Yes	Applicable to the mentioned above.	Applicable to the mentioned above

Online path searching based on the sampling roadmap is an important process. Yuan et al. (2015) proposed a hybrid sampling strategy composed of bridge test sampling and non-uniform sampling to increase the number of effective sampling points in narrow corridors and boundary regions to generate a roadmap. Then the optimized A* algorithm is applied to search the path with redundant edges removal. Hrabar (2008) proposed a combination of the PRM algorithm and D* Lite for path planning, where a stereo camera embedded in the robot is used to detect obstacles and dynamically update the path in unknown configuration space. Khaksar et al. (2019) also proposed a combination of the D* Lite algorithm and random roadmap algorithm for path planning in complex terrain.

In general, there is not much work to deal with the issue of generating effective sampling points in narrow corridors, though the path planning with sparse obstacles has been extensively discussed (Qureshi and Ayaz, 2016). Additionally, most search-based path planning methods consume a lot of memory and time to find the optimal path, while they are quite sensitive to the global environment information as well. This article aims to propose a sampling strategy based on obstacle potential field to tackle this issue. Considering the potential of the obstacle configuration space to negotiate with the reactive robot, sampling is only performed in the obstacle area and its vicinity. Each sampling point is connected with the nearest vertices, and the edge intersecting with the obstacle is removed by collision detection so as to construct a feasible and optimal roadmap. The contributions of the article are summarized as follows:

1. By introducing the potential field, an effective sampling strategy is proposed to determine a certain range around the obstacle boundary as the specific target sampling area. For each sampling point, an edge connection is made with the nearest vertices adjacent to each other, while the edges that intersect with the obstacle are removed. Hence, an effective roadmap can be constructed with minimum feasible sampling points.
2. The upper limit of the potential field is proposed to tackle the mobile obstacles so as to reduce the load of path replanning and enhance the online path planning efficiency and adaptability.
3. Combined with the constructed roadmap, an incremental D* Lite search algorithm is adopted to dynamically search the collision-free path online, which has higher real-time performance than that of the traditional A* algorithms.
4. Extensive experiments have been performed to testify to the effectiveness of the proposed method with the comparison of the current path planning algorithms.

The remainder of the article is organized as follows. The roadmap construction for the path planning is explained in Section 2. Section 3 introduces the proposed path planning algorithm including potential-based sampling generation and the involved strategy. In Section 4, the experiments are performed to verify the effectiveness of the proposed path planning algorithm, and comparison experiments are also provided with typical sampling-based algorithms. The conclusion is given in Section 5.

## 2. Roadmap Construction Preparation

The roadmap construction is fundamental to available path searching in the configuration space, while the roadmap method performs well with light computational cost in robot motion planning (Lin and Saripalli, 2017), i.e., PRM. Here, for sampling, only a straight line connection is considered.

Probabilistic Roadmap Method requires traversing all the queues of the collision twice when constructing the roadmap in *C*_*free*_, where the roadmap construction process is described with Algorithm 1. First, for each random sampling, it is necessary to detect whether it falls into *C*_*obst*_ and perform collision detection for all sampling points (Referring to line 5 in Algorithm 1). Second, each sampling point in the vertex set *V* is connected by a straight line according to a local search algorithm, such as k-nearest, and all edge queues are traversed to remove the edges that intersect the obstacle area (Referring to line 12 in Algorithm 1). Obviously, this kind of roadmap has poor connectivity in narrow corridors due to its small proportion of the configuration space. The key to solving this connectivity problem is to generate more effective sampling points inside the narrow corridors.

**Algorithm 1:**
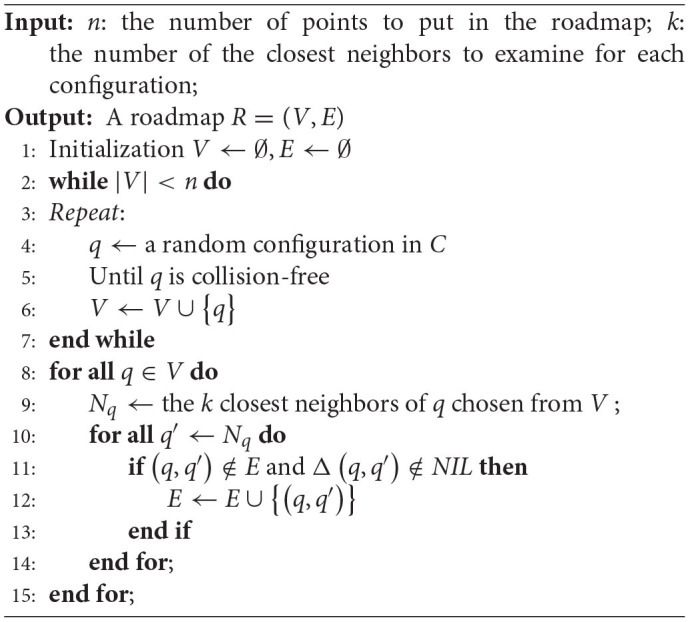
Roadmap Construction Algorithm.

It should be mentioned that narrow corridors usually exist among obstacle configuration spaces, while the smaller the width of the corridor, the tighter they surround the obstacles. If the sampling in *C*_*free*_ is constrained to only sampling in a certain range around the obstacle area, sampling points could effectively fall into the narrow corridors between obstacle configuration spaces. Hence, a sampling strategy is proposed to avoid motion assumptions and heavy calculation.

As it is known, a roadmap is a collection of edges determined by two feasible points with obstacles avoidance, where the key to roadmap construction is to use effective sampling points as much as possible. Here, on the inspiration of Qureshi and Ayaz (2016), a potential field is introduced in the configuration space used to assist sampling so as to increase the number of effective sampling points in the narrow corridors among the obstacle configuration spaces. The involved sampling generation will be explained in the next section.

## 3. The Proposed Algorithm

The proposed online path planning algorithm is explained in detail.

### 3.1. Potential Field Based Sampling Strategy

The potential field is a potential energy field artificially constructed using a gradient descent method (Khatib, 1986). In the potential field, the obstacle is assigned with repulsive potential *U*_*rep*_, causing the robot to be repulsed by the obstacle. The applied robot is assumed as a particle, without shape and dynamics consideration due to simplicity, since the path planning could be irrelevant to the robot itself (Kantaros and Zavlanos, 2016). The target area is assigned with the attraction potential *U*_*att*_, and the robot tends to be attracted to the target. The repulsive and attractive potentials can make the force *F* that the robot bears equal to the negative gradient of the electric potential, i.e., *F* = −∇*U*, where *U* represents the superposition of *U*_*rep*_ and *U*_*att*_ and ∇ is the differential operator. The robot would move along the direction where the gradient drops the fastest without collision.

The shortest distance *d*_*min*_, from the current position to the closest vertex in *C*_*obst*_ is calculated as,


(1) $$\begin{array}{r} d_{\min}=\underset{q^{\prime }\in C_{obst}}{\min}d\left(q,q^{\prime }\right) \end{array}$$

where $q^{\prime }\in C_{obst}$ is the obstacle state closest to the current state *q* ∈ *C*_*free*_ and *d*(*q, q*′) is the straight line distance between the two points, *q* and *q*′. The repulsive potential generated in *C*_*obst*_ is represented as,


(2) $$U_{rep}=\{\begin{array}{cc} \frac{1}{2}K_{r}{\left(\frac{1}{d_{min}}-\frac{\text{1}}{d_{obst}^{*}}\right)}^{2} & d_{min}\leq d_{obst}^{*}\\ 0 & d_{min}>d_{obst}^{*} \end{array}$$

where *K*_*r*_ is the gain coefficient proportional to the magnitude of the repulsive potential defined by the original potential field principle. If the distance *d*_min_ is greater than the constant $d_{obst}^{*}$, the repulsive potential of the robot from the obstacle is considered to be zero, which indicates that the robot is far away from the nearest obstacle area.

The repulsive force generated by the obstacle repulsive potential is given as Equation (3), which is equal to the negative gradient of the repulsive potential in Equation (2),


(3) $${\vec{F}}_{rep}=\{\begin{array}{cc} K_{r}(\frac{\text{1}}{d_{obst}^{*}}-\frac{1}{d_{min}})\frac{1}{d_{min}^{2}}\frac{\partial d_{min}}{\partial q} & d_{min}\leq d_{obst}^{*}\\ 0 & d_{min}\geq d_{obst}^{*} \end{array}$$

where $\frac{\partial d_{\min}}{\partial q}=\frac{\left(q-q^{\prime }\right)}{d\left(q,q^{\prime }\right)}$. Both repulsive potential and repulsive forces decrease gradually from the obstacle area to the outside area along the gradient.

**Figure 1:**
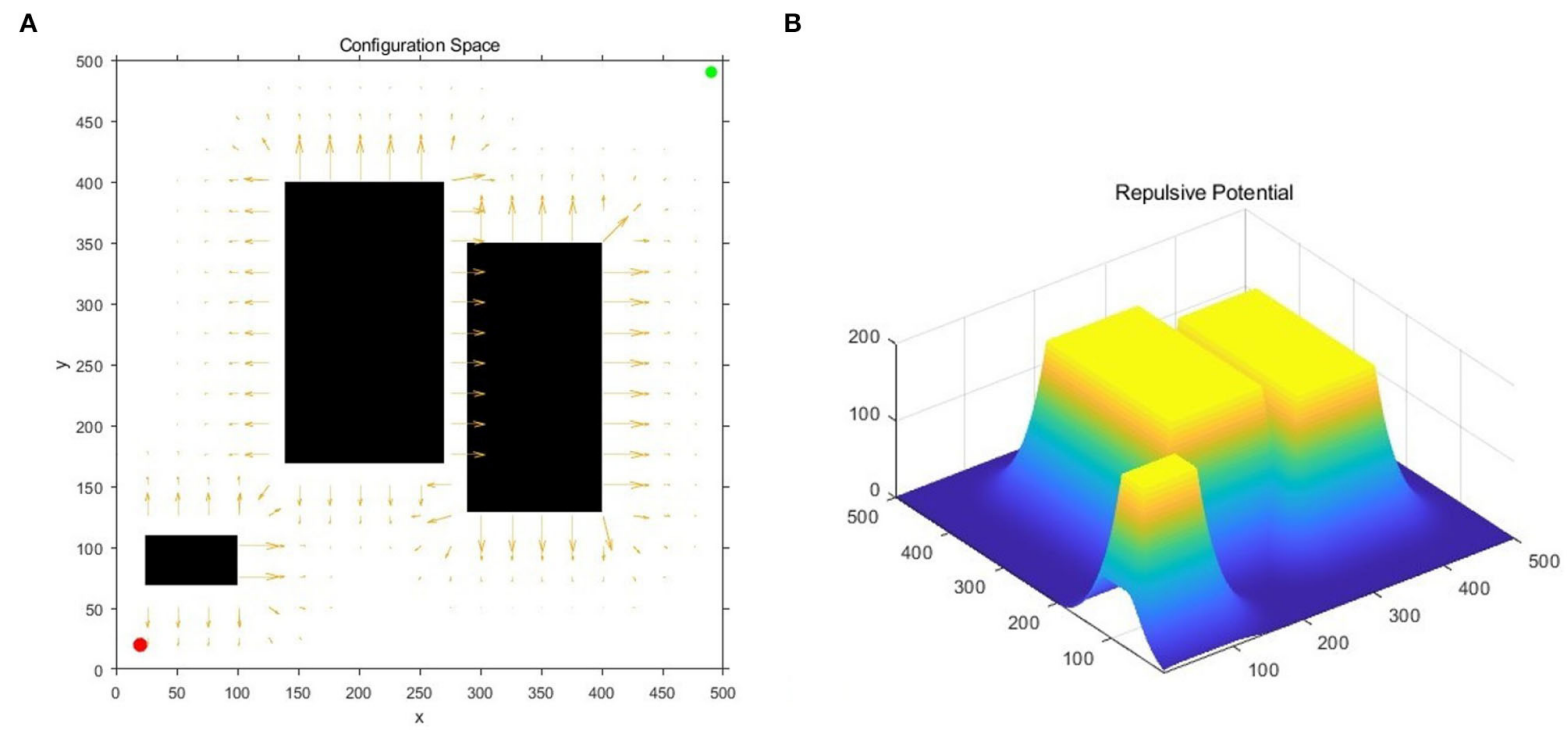
Obstacle repulsive potential field experienced by random samples: **(A)** The diagram of the potential field direction; **(B)** The diagram of the potential field gradient.

Due to the existence of the repulsive potential in the obstacle area, the obstacle has a certain potential energy impact on the surrounding free configuration space, and diffuses to the surroundings in the form of a potential field gradient, as shown in Figure 1. In detail, red and green dots represent the starting and target points of the robot, black areas represent obstacles, and arrow rays represent the potential field generated by the obstacle configuration space in Figure 1A. Additionally, in Figure 1B, the vertical and horizontal correspond to the potential and position, respectively.

For narrow corridor sampling, this gradient can help determine the boundary of the obstacle and sampling near the boundary of the appropriate range, so as to increase the number of sampling points falling into the feasible corridor. Algorithm 2 describes the process of using gradient descent in the potential field, where λ is a small incremental distance. According to the gradient, the minimized boundary range of the obstacles can be easily calculated including narrow corridors, and the appropriate gradient range can be intercepted by the density of obstacles in the configuration space.

**Algorithm 2:**
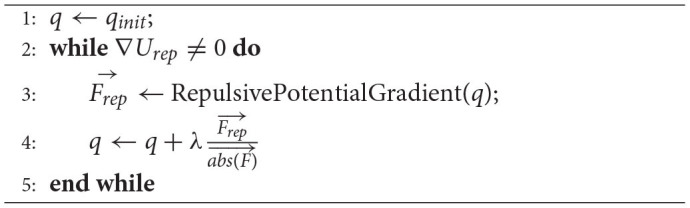
GradientDescent(*q_init_*).

In this article, a simple but effective method is adopted to determine the obstacle boundary and target sampling space by introducing potential fields into the planning configuration space. First, the potential field of each sampling point is calculated to ensure the connection of the narrow corridors. The potential field set threshold is defined as,


(4) $$\begin{array}{r} U_{0}=U_{rep}-\lambda \frac{\vec{F_{rep}}}{\vec{abs\left(F\right)}} \end{array}$$

**Figure 2:**
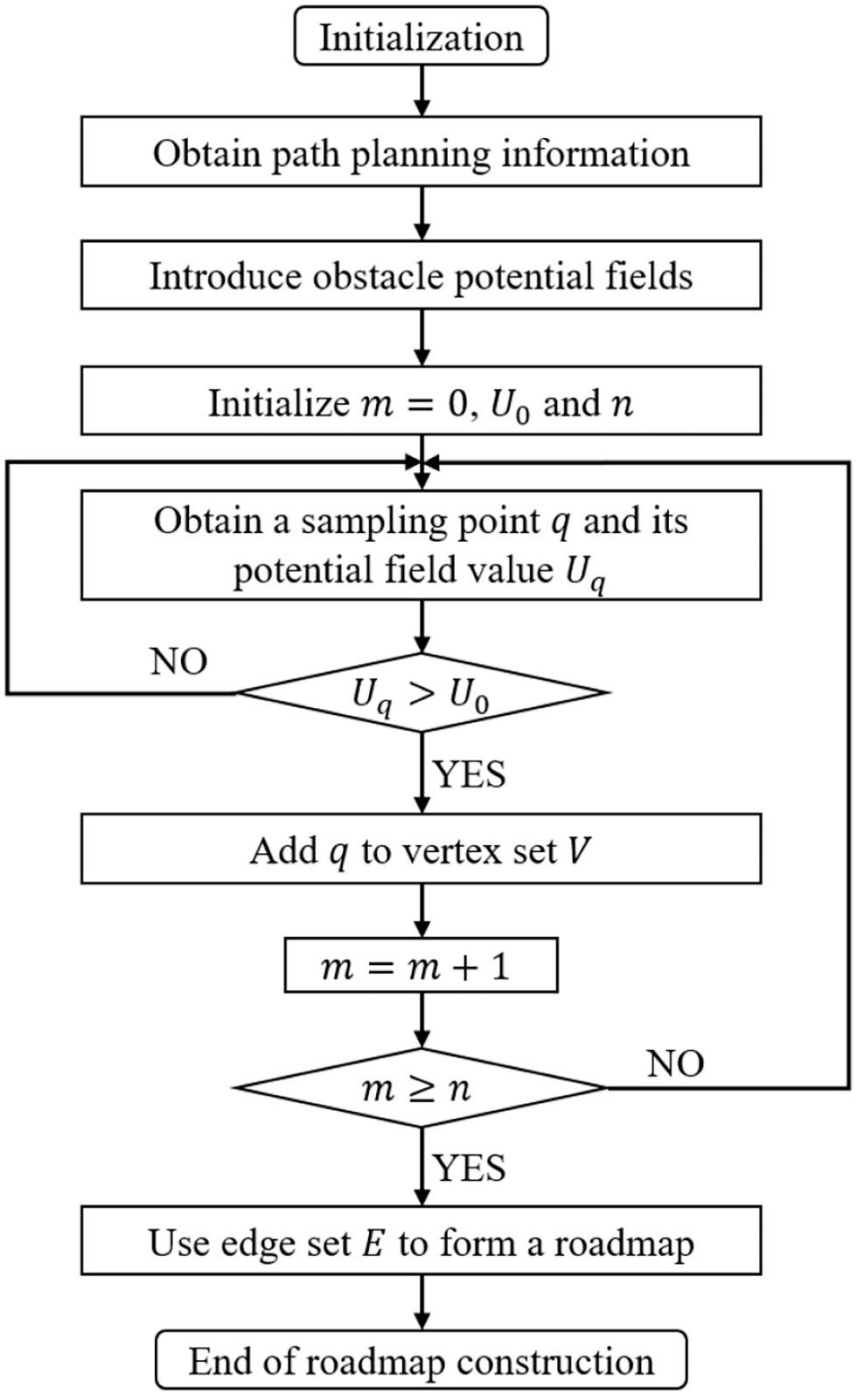
Roadmap construction based on obstacle potential field.

The potential field value *U*_*q*_ of the sampling points which is larger than the potential field set threshold is saved in the vertex set list. In other words, only samples around the obstacle boundaries are selected. The whole algorithm design process is shown in Figure 2.

This improved algorithm is called OP-PRM (Obstacles Potential field-Probabilistic Roadmap Method), as the PRM is regarded as the basic sampling algorithm whose original sampling strategy would be replaced by the proposed strategy based on the potential field. The 1st step is to initialize and obtain the path planning information, including starting point, target point, and target planning configuration space. The target planning configuration space includes *C*_*free*_ and *C*_*obst*_.

In the 2nd step, artificial potential fields are introduced into the configuration space, considering the narrow corridor barriers between the obstacle configuration spaces, for the purpose of increasing the number of sampling points falling in the corridors as much as possible. The 3rd step is to compare the potential field value *U*_*q*_ of the sampling point with the set value *U*_0_. If *U*_0_ ≤ *U*_*q*_, more sampling points closer to the obstacle configuration space are added in the vertex set *V*, and vice versa.

Then the 3rd step is repeated until the number *k* of sampling points reaches the threshold. Thus, a roadmap can be connected, though the constructed network map may partially pass through the obstacle configuration space. After the collision detection process, the edges that intersect the obstacle are removed, and the routes near the obstacle configuration space are still retained to improve the connection feasibility of the narrow passage.

In general, the major difference between the OP-PRM and the PRM is that the sampling points in the obstacle configuration space are considered during the sampling process, but the range of the obstacle configuration space is expanded according to the repulsive potential field gradient while narrow corridors are included as well. On the other hand, the sampling points close to the obstacle boundary are retained to establish more potentially available shorter paths.

**Figure 3:**
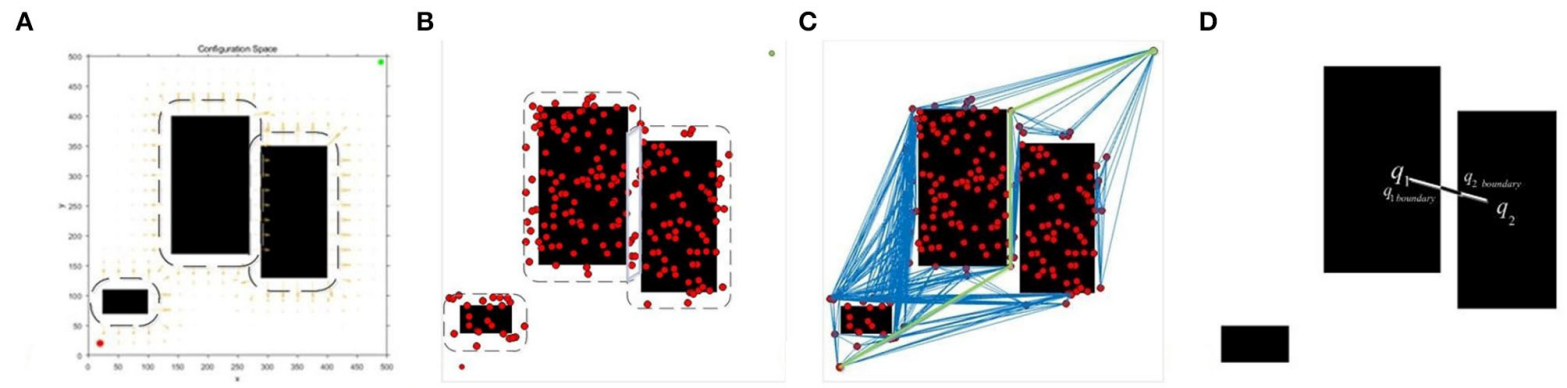
Sampling strategy based on the obstacle potential field: **(A)** Obstacle potential field; **(B)** Target sampling result; **(C)** Random roadmap; **(D)** Narrow corridor width calculation.

The key to sampling strategy based on obstacle potential field is how to determine the target sampling area, which is shown in Figure 3. The dotted area in Figure 3A represents the target sampling area determined by the obstacle potential field, and the red dots in Figure 3B represent the pending vertices randomly falling into the target area of the obstacle potential field. When the potential fields of two obstacles expand from the boundary to the periphery, the two potential field areas are overlapped in the narrow corridor. The optimal range is that the potential field of the one obstacle just extends to the nearest boundary of the other obstacle without touching the other obstacle. Based on the sampling strategy of obstacles, samples near the obstacle will be selected and the sampler usually discards the sampling points that fall into the obstacle area. With the constructed roadmap, only the edges that intersect the obstacle are discarded *via* OP-PRM, as shown in Figure 3C, while the connection between the vertices is represented by the blue lines.

As shown in Figure 3D, *q*_1_ moves toward *q*_2_ along the connected straight line of the two points until reaching the boundary of the obstacle so as to acquire the boundary point *q*_1*boundary*_, similarly to *q*_2_ to obtain the boundary point *q*_2*boundary*_. Therefore, the width of the narrow corridor can be calculated as,


(5) $$\begin{array}{l} \begin{array}{ccc} d & = & \sqrt{{\left(x_{q1boundary}-x_{q2boundary}\right)}^{2}+{\left(y_{q1boundary}-y_{q2boundary}\right)}^{2}} \end{array}\\ \;\begin{array}{c} \cdot \frac{abs(x_{q2}-x_{q1})}{\sqrt{{\left(x_{q2}-x_{q1}\right)}^{2}+{\left(y_{q2}-y_{q1}\right)}^{2}}} \end{array} \end{array}$$

Then the coefficient λ can be obtained as,


(6) $$\begin{array}{r} \text{λ}=kd \end{array}$$

where *k* is a proportional constant whose value is less than 1 to ensure that a safe distance is reserved around the obstacles. Given the width of the narrow corridor, the threshold value of the potential field can be obtained *via* Equation (4). The sampling area based on the obstacle potential field is determined by the threshold value, i.e., *U*_*q*_ ≥ *U*_0_.

### 3.2. Search Strategy

In the random roadmap, the priorities of the connected vertices are evaluated as,


(7) $$\begin{array}{r} f\left(q\right)=g\left(q\right)+h\left(q\right) \end{array}$$

where *g*(*q*) is the actual cost from the current point to the starting point and *h*(*q*) is a heuristic function, representing the estimated value from point *q* to the target point. As a result, *f*(*q*) is the priority evaluation value of the vertex *q*, and the smaller the *f*(*q*) value, the higher the priority of the vertex.

Compared with the A* algorithm, the D* Lite algorithm can dynamically search the optimal path in real-time. Therefore, the incremental heuristic D* Lite algorithm is used here. The main idea of the D* Lite algorithm is to search the reverse path from the target point to the starting point when a new obstacle is found in the path so that the corresponding heuristic function *h*(*q*) becomes the estimated value from the point *q* to the starting point, and *g*(*q*) is the cost from the point *q* to the target point. When a new obstacle is detected, the D* Lite algorithm does not need to completely replan the path, while the original information can still be used to find a path with obstacle avoidance. The D* Lite method is given in Algorithm 3 and readers who are interested in the algorithm can refer (Koenig and Likhachev, 2005). Note that heuristic solutions are mostly near-optimal solutions with the trade off computational efficiency.

**Algorithm 3:**
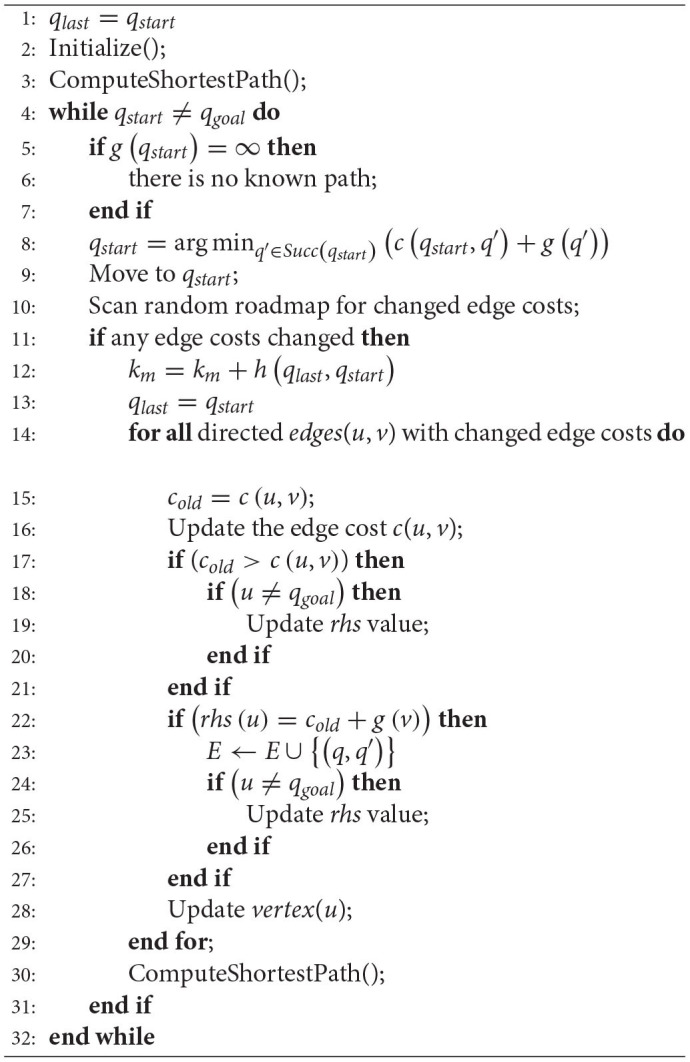
Main program of D* Lite.

### 3.3. Dynamic Obstacle Accommodation

**Figure 4:**
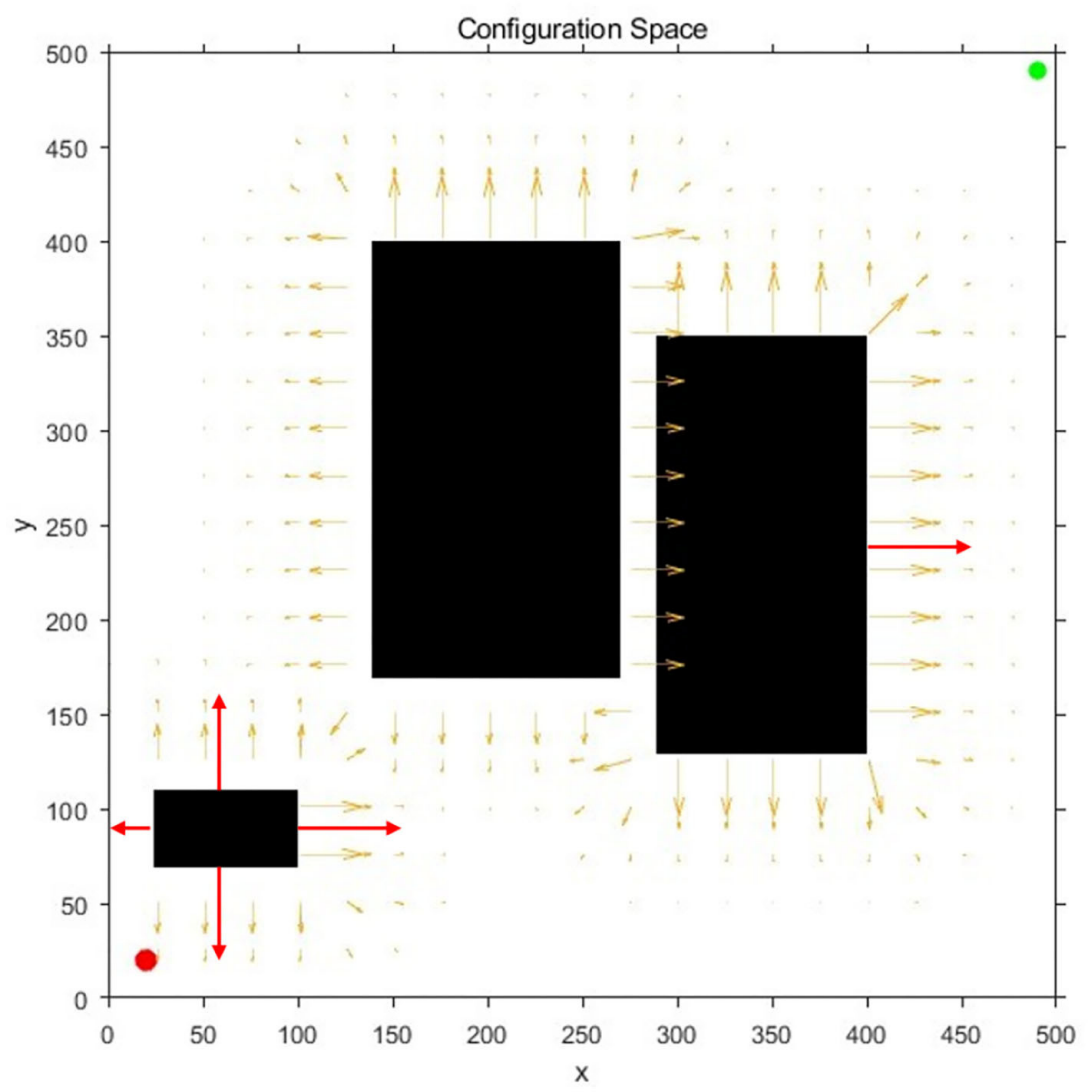
An example of the combination of obstacles moving direction. The red arrows represent the moving directions of the obstacles.

The proposed path planning algorithm is also able to dynamically search the optimal path with the assistance of previous searched information when the obstacles move within a certain distance in an omni direction, which is demonstrated in Figure 4.

Let *U*_*rep*_ and *U*_*att*_ be the potentials of one sampling point before the obstacles move and Δ*U*_*att*_ and Δ*U*_*rep*_ be the potential differences. The ranges of new potential field values are derived as,


(8) $$\left\{ \begin{array}{cc} \begin{array}{c} U_{upper}=\frac{U_{att}^{2}}{\min(U_{att})}+U_{rep}\\ U_{lower}=\frac{U_{att}^{2}}{\max(U_{att})}+U_{rep} \end{array} & if\;\;\text{Δ}U_{att}\geq \text{Δ}U_{rep} \end{array}\right.$$

(9) $$\left\{ \begin{array}{cc} \begin{array}{c} U_{upper}^{{}^{\prime }}=\frac{U_{rep}^{2}}{\min(U_{rep})}+U_{att}\\ U_{lower}^{{}^{\prime }}=\frac{U_{rep}^{2}}{\max(U_{rep})}+U_{att} \end{array} & if\;\;\text{Δ}U_{att}<\text{Δ}U_{rep} \end{array}\right.$$

where min(*U*_*att*_) and min(*U*_*rep*_) represent the minimum values of the attractive potentials and repulsive potentials corresponding to all points within the neighborhood of the current location, and max(*U*_*att*_) and max(*U*_*rep*_) represent the maximum values of the attractive potentials and repulsive potentials, i.e., the potential of the points within the neighborhood of the current location is calculated and compared to obtain the maximum and minimum, respectively. The scope of the neighborhood depends on the tolerance to environmental variation. The ranges of the new potential field values can be divided into two cases:

1. If the attractive potential difference is larger than the repulsive one, the attractive one would be the main factor affecting the range;
2. If the repulsive one is larger, it would play a more vital role in the range determination.

Hence, once the obstacles in the planning configuration space have moved and the new potential field values are derived from the superposition of new attractive potentials and repulsive potentials, the strategy used here is described as follows. If the new potential field value is within the range in Equation (8) or Equation (9) and the direction of the potential field is the same as that of the last moment, it means that the movement of the obstacles is within the tolerance which has not reversed the direction of the potential field, the previous searched information would be adopted for path planning; Otherwise, the path should be replanned. In a such case, the proposed OP-PRM path planning strategy can accommodate mobilized obstacles in a certain range with more adaptivity and higher efficiency.

## 4. Experiment Result and Analysis

In this section, experiments for the path planning of the robot are demonstrated. In the simulation experiments, the kinematics and dynamics of the robot are not considered, where the robot is regarded as a particle and the performance of the proposed OP-PRM algorithm is verified *via* MATLAB simulation.

**Figure 5:**
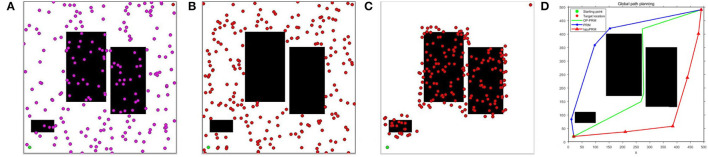
The sampling results of lazyPRM, PRM, OP-PRM algorithms at *n* = 200: **(A)** LazyPRM sampling process; **(B)** PRM sampling process; **(C)** Proposed OP-PRM sampling process; **(D)** Obtained path planning result.

The sampling processes of the lazyPRM, PRM, and OP-PRM algorithms are demonstrated in Figures 5A–C. It can be seen that the LazyPRM algorithm samples the entire map with no collision detection, hence the sampling is uniformly random. PRM algorithm includes collision detection of sampling points in the sampling process, so there is no sampling point in the obstacle area which is also uniformly random sampling in the free area. Although the OP-PRM algorithm does not have collision detection during the sampling process, with the introduction of the obstacle potential field, the sampling is only performed around the obstacles and their vicinities, and the sampling randomness can be reduced as a regional heuristic sampling. In comparison with the sampling efficiency of the three algorithms, when *n* = 100, *n* = 200, and *n* = 300 (where *n* is the number of sampling points in each experiment), each algorithm is sampled 100 times and the average values of the sampling points in the narrow channel are calculated, summarized in Table 2.

**Table 2:** Performance evaluation of the path planning algorithms with 100 experiments.

**Experiment category**	**Sampling points/Algorithm**	***n* = 100**	***n* = 200**	***n* = 300**
The average number of sampling points in a narrow corridor	LazyPRM	1.2	2.5	3.5
	PRM	1.65	2.55	3.15
	Proposed OP-PRM	3.6	6.9	10.4
The success rate of passing through the narrow corridor	LazyPRM	85%	91%	93%
	PRM	83%	92%	95%
	Proposed OP-PRM	80%	90%	95%
The times of planned path through the narrow corridor	LazyPRM	4	9	12
	PRM	19	27	38
	Proposed OP-PRM	75	86	90
The average distance of the path planned (pixels)	LazyPRM	791.94	782.62	776.72
	PRM	770.05	772.28	750.99
	Proposed OP-PRM	761.44	745.68	740.98
The average execution time of path planning (seconds)	LazyPRM	0.98	3.87	8.23
	PRM	7.52	29.27	65.69
	Proposed OP-PRM	1.16	3.74	8.41

**Figure 6:**
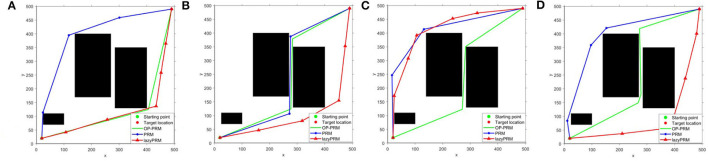
**(A–D)** The comparison of global path planning with LazyPRM, PRM, and OP-PRM algorithms.

It can be seen that the proposed OP-PRM algorithm can improve the number of sampling points in the narrow channel with different *n* values compared to PRM and LazyPRM, and can also improve the sampling efficiency in the narrow channel with a smaller number of sampling points. Under the same condition, a group of path planning experiments is conducted to search for the optimal path from the starting point to the target point. In the two-dimensional configuration space, the maximum value of the sampling point is set to *n* = 200, and the result of global path planning of the three algorithms is shown in Figure 5D where only the robot accompanied by the OP-PRM algorithm succeeds in passing through the narrow corridor. Furthermore, other cases of global path planning with the three algorithms are depicted in Figure 6, as the extension of Figure 5D. The comparison of Figures 6A,C,D shows that the robot accompanied by the OP-PRM algorithm can pass through the narrow corridor in most cases which are declared in Table 2, while Figure 6B is the case where both the robot applied with PRM and OP-PRM algorithm succeeds in passing through the narrow corridor.

**Figure 7:**
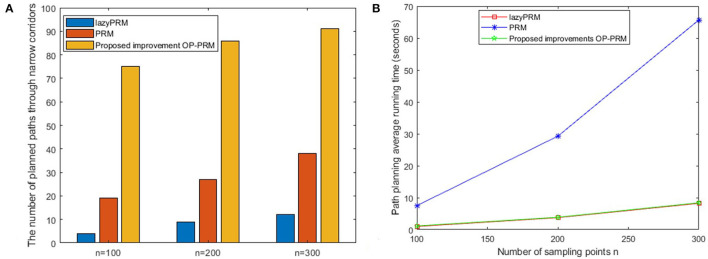
The Comparison of experimental results with the criteria of passing time and execution time: **(A)** The number of the planned paths through narrow corridors; **(B)** The average execution time of the path planning.

After 100 sets of independent experiments, the number of times the LazyPRM, PRM, and OP-PRM algorithms pass through the narrow channel is shown in Figure 7A. It can be seen that the OP-PRM algorithm can improve the success rate of passing through narrow channels under different sampling points.

The execution time is also an important criterion for the performance evaluation of path planning. The execution time of the three algorithms at 100 sets of different *n* values is averaged, as shown in Figure 7B. The LazyPRM algorithm is not applied to perform collision detection on sampling points based on the basic PRM algorithm, since collision detection on all sampling points is a time-consuming process. The OP-PRM algorithm in this article also does not perform the collision detection process, so its execution time is almost the same as that of the LazyPRM, and the sampling area is heuristically limited to the obstacle area and its surroundings.

Furthermore, when the upper limit of the number of sampling points is *n* = 300, the execution time of the lazyPRM and the OP-PRM does not exceed 10 s, while the execution time of the PRM is about six times more than the other two algorithms, which identify that performing no collision detection on the sampling process can save much time. Moreover, the PRM algorithm requires performing collision detection at each sampling point during the sampling process, so the execution time would increase greatly as the number of samples increases.

**Figure 8:**
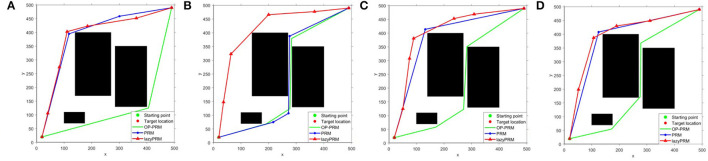
**(A–D)** The experiments of dynamical obstacle accommodation.

When the obstacles move within a certain distance, the path planning results of the OP-PRM, PRM, and LazyPRM algorithms are shown in Figure 8. In this group of experiments, compared with the position of the obstacles in Figure 6, the bottom obstacle here has moved 70 pixels toward the right direction and the results are similar to Figure 6. The RRTs (Ferguson et al., 2006; Zucker et al., 2007), RRTX (Otte and Frazzoli, 2016), and the replanning methods based on RRT (Bekris and Kavraki, 2007) would require performing rewire operations of the cascade connection of the affected branches to repair the graph and remodel the shortest-path tree even with slightest obstacle modification detection, while our proposed method can accommodate roughly large obstacle movement without replanning.

**Figure 9:**
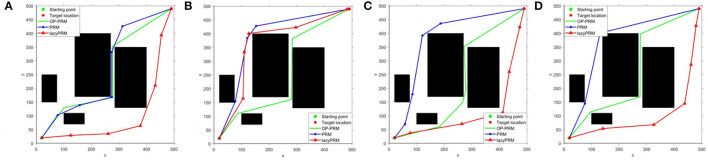
**(A–D)** Different scenarios with more generated obstacles to testify the dynamical ability of the OP-PRM.

Results in different scenarios to testify the ability to plan with dynamic obstacles are depicted in Figure 9, where the bottom obstacle has moved 70 pixels toward the right direction and there is one more obstacle on the far-left side compared with the configuration space in Figure 6. Both Figure 9 demonstrate that our proposed algorithm can deal with the mobile obstacles during real-time path planning. In specific, the rules we have indicated in Dynamic obstacle accommodation could handle the slight obstacle movement without re-sampling and roadmap update, and the sampling and roadmap would update only if the obstacles move greatly.

**Figure 10:**
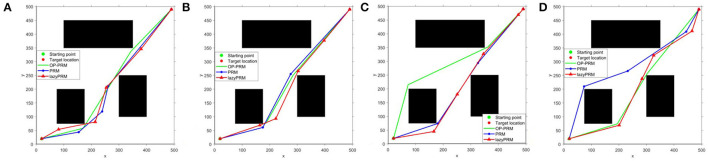
**(A–D)** The performance comparison in the environments without a narrow corridor.

**Figure 11:**
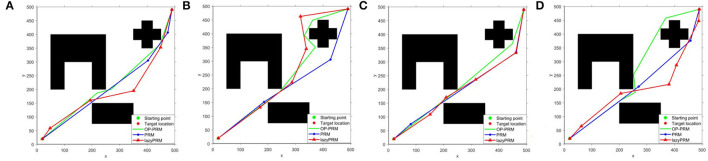
**(A–D)** The performance comparison in environments with concave polygon obstacles.

In addition, the performance comparison of lazyPRM, PRM, and OP-PRM algorithms in environments without narrow corridors is performed to show their capability with sparse obstacles, where the vertical and horizontal distances among obstacles exceed 100 pixels, as shown in Figure 10. The three algorithms share almost the same path in Figures 10A,B, while the planned path with OP-PRM is the longest in Figure 10C and shortest in Figure 10D, i.e., the OP-PRM shows certain varied capacity due to the randomness of its sampling point selection. Furthermore, considering more complex cases, i.e., the environments with concave polygon obstacles, our proposed method can transform the concave polygon obstacles into convex polygon ones by connecting their vertices as a path to adapt, while the four subfigures (refer to Figure 11) demonstrate different ways of passing through the narrow corridor. In these environments, OP-PRM and PRM share similar paths, while the planned paths with PRM and OP-PRM are the shortest among the three algorithms in Figures 11B,C. On the other hand, the result of Figure 11D can illustrate the limitation of the proposed sampling strategy, as the OP-PRM would generate some sampling points close to the leftmost obstacle in this situation, leading to a longer path. It should also be noted that the average execution time in the experiments with concave polygon obstacles is 2.8s, 13s, and 6.1s for OP-PRM, PRM, and LazyPRM, respectively, which once again verifies the effectiveness and efficiency of the proposed method.

To summarize, the performance of the proposed OP-PRM algorithm is compared with the LazyPRM and the PRM algorithms *via* the number of sampling points in the narrow corridor, the execution time, and path length of the path planning aspects. The sampling efficiency of the OP-PRM algorithm in narrow corridors is significantly higher than those of the two algorithms, while the execution time of the OP-PRM is almost the same as the LazyPRM, both of which are several times faster than that of the PRM. The average path distance of the OP-PRM is the shortest as well. Hence, the proposed OP-PRM has higher online path planning performance, especially in dynamical obstacle environments. Although the proposed method is discussed in 2D maps, it can be extended to 3D space with the potential field principle for obstacle detection and available free nearest path generation. It is further mentioned that the performance of the effective sampling points with the proposed method falling into the narrow corridor is not affected by the width of the corridor, since the repulsive forces of the obstacles are irrelevant to the distance between the obstacles. Moreover, only comparisons are made among PRM, LazyPRM, and the proposed method, for which we are concerned is the effectiveness of the sampling strategy with the introduction of the potential field to the narrow corridor.

## 5. Conclusion

This article proposes a probabilistic roadmap algorithm based on the obstacle potential field sampling strategy, called the Obstacle Potential field Probabilistic Roadmap method (OP-PRM). The obstacle potential field is introduced to determine the obstacle area and a certain range near the obstacle boundary as the target sampling area. This new method can increase the number of effective sampling points that fall into narrow corridors in a simple and efficient way so as to construct a concise connected random roadmap, even under mobile obstacles conditions. Furthermore, the incremental heuristic D* Lite algorithm is applied to search for the shortest paths between the starting point and the target point on the roadmap. A two-dimensional map simulation experiment has been performed to demonstrate that the OP-PRM path planning algorithm can allow the robot to pass through the narrow corridor map with a faster speed and higher success rate.

Further research will extend the proposed algorithm to a variety of complex maps and three-dimensional environments so as to enhance flight autonomy.

## Data Availability Statement

The original contributions presented in the study are included in the article/supplementary material, further inquiries can be directed to the corresponding authors.

## Author Contributions

All authors listed have made a substantial, direct, and intellectual contribution to the work and approved it for publication.

## Funding

This study was supported under the National Key Research and Development Program of China (2018YFB1305505), National Natural Science Foundation of China (NSFC) (61973296 and U1913201) STS project of the Chinese Academy of Sciences (KFJ-STS-QYZX-107), Shenzhen Science and Technology Innovation Commission Project Grant (JCYJ20170818153635759), and Shenzhen Fundamental Research Program (JCYJ20200109114839874).

## Conflict of Interest

The authors declare that the research was conducted in the absence of any commercial or financial relationships that could be construed as a potential conflict of interest.

## Publisher's Note

All claims expressed in this article are solely those of the authors and do not necessarily represent those of their affiliated organizations, or those of the publisher, the editors and the reviewers. Any product that may be evaluated in this article, or claim that may be made by its manufacturer, is not guaranteed or endorsed by the publisher.
